# Dr. R. D. Arnold, of Savannah

**Published:** 1876-08

**Authors:** 


DR. R. D. ARNOLD, OF SAVANNAH.—We are called upon to record the death of this great and good man. He died on the 10th of July, 1876, aged 68 years, of acute pulmonary phthisis. We have received the following notice of his death and short sketch of his life and labors from those who have known him long and well. We can but add our profound sorrow:

"At a meeting of the Georgia Medical Society, held on Wednesday, the 19th instant, the following report of the committee appointed for the purpose was adopted:

After an illness of several weeks' duration, Dr. Richard Arnold died on the 10th instant, in the 68th year of his age, of acute pulmonary phthisis.

His death, though not unexpected, produced a profound sense of sorrow, and the spontaneous gathering of the whole community at his obsequies attested the appreciation in which he was held as the kind and skillful physician as well as the intelligent and faithful public citizen.

After a thorough preparatory course of literary and scientific study at Princeton, New Jersey, he received the degree of Doctor of Medicine from the Medical Department of the University of Pennsylvania in 1830, at that time the foremost school of medicine in this country, and soon afterwards commenced the active duties of his profession in his native city.

Earnest in his efforts for the acquisition of knowledge, possessing a mind with keen perceptive faculties and an extraordinarily retentive memory, he soon attained a prominent position in his profession.

Kind and charitable to the poor, attentive and faithful in in the performance of his professional duties, and with high social qualities, he soon gained a place in the esteem and affections of his fellow-citizens, which he continued to hold until the day of his death.

Appointed in 1835 one of the physicians of the Savannah Poor House and Hospital, which appointment was renewed annually for more than thirty years, he acquired a perfect familiarity with the diseases of our climate, and his published monograph on bilious and yellow fevers has made him an authority on those subjects, which is recognized by the best writers in the country. He was a member of the American Medical Association from its inception in 1846, and co-operated heartily in the objects of its formation. He was one of the committee that framed the "Code of Ethics" by which the whole medical profession in the United States is governed, and at its fourth annual meeting, held in Charleston, S. C., in 1851, was elected one of its Vice-Presidents.

Upon the recommendation of the Association the State Medical Societies should be formed as auxiliaries in the great work of medical reform, Dr. Arnold took an active part in the organization of the Medical Society of the State of Georgia, and as President in 1851, in Atlanta, delivered an able address upon "Reciprocal Duties of Physicians and of the public towards each other," in which he advocated a more thorough preparatory course of instruction in English, Greek and Latin literature, as well as the collateral sciences, before commencing the study of medicine.

Upon the organization of the Savannah Medical College in 1850, he became Professor of the Theory and practice of Medicine, and was thus enabled to lay before his classes the vast treasures of learning which he had gathered from reading and experience.

His manner of imparting knowledge was plain and lucid, and he always preferred to illustrate his teachings by clinical instruction, which his connection with the Savannah Hospital enabled him easily to do.

In the beginning of his medical career in Savannah he became connected with the Georgia Medical Society, and was ever an active and efficient member. Upon the death of Dr. Posey in 1860 he was elected President and continued in that office until the annual election in 1868. In consequence of the troubles of the country, and especially during the stirring events which occurred between 1861 and 1865, necessitating the absence from the city of many of its members, the Georgia Medical Society had but few meetings—and those very sparely attended—but in 1865 the society revived, and Dr. Arnold rarely absented himself from its weekly meetings unless compelled by pressing professional or other duties.

An earnest worker in the cause of medical education, he endeavored to elevate the standard of the profession by lengthening the curriculum of study and demanding a more thorough preparatory training in elementary branches.

As an advocate of various sanitary measures, his efforts in securing the efficiency and fostering the system of dry culture, for procuring an ample supply of pure water, and for a thorough system of drainage, have resulted in making the city of Savannah one of the healthiest, not only in the South, but in the whole country, and are a proud monument to his intelligence and patriotism.

In his intercourse with his professional brethren he was high-toned, honorable, generous—but no man looked upon anything having the slightest appearance of charlatanism or quackery with greater scorn and disgust than Dr. Richard D. Arnold.

Your committee recommend the adoption of the following resolutions:

*Resolved.* That the members of the Georgia Medical Society most sincerely lament the loss of one of our most faithful, intelligent and valuable members—one whom we admired for his extensive mental acquirements, esteemed for his manly virtues, and loved for his social qualities.

*Resolved* 2d. That we deeply sympathize with his afflicted family in their sad bereavement.

*Resolved* 3d. That this report be spread upon the minutes and a copy of the same be transmitted to the family of Dr. Arnold.

EASTON YONGE, M.D.
W. M. CHARTERS, M.D.
ROB. P. MYERS, M.D.
Committee.

*Savannah, Ga., July* 19, 1876.

